# Novel Luminex Assay for Telomere Repeat Mass Does Not Show Well Position Effects Like qPCR

**DOI:** 10.1371/journal.pone.0155548

**Published:** 2016-05-16

**Authors:** Muhammad G. Kibriya, Farzana Jasmine, Shantanu Roy, Habibul Ahsan, Brandon L. Pierce

**Affiliations:** Department of Public Health Sciences, Biological Science Division, The University of Chicago, Chicago, Illinois, United States of America; Tulane University Health Sciences Center, UNITED STATES

## Abstract

Telomere length is a potential biomarker of aging and risk for age-related diseases. For measurement of relative telomere repeat mass (TRM), qPCR is typically used primarily due to its low cost and low DNA input. But the position of the sample on a plate often impacts the qPCR-based TRM measurement. Recently we developed a novel, probe-based Luminex assay for TRM that requires ~50ng DNA and involves no DNA amplification. Here we report, for the first time, a comparison among TRM measurements obtained from (a) two singleplex qPCR assays (using two different primer sets), (b) a multiplex qPCR assay, and (c) our novel Luminex assay. Our comparison is focused on characterizing the effects of sample positioning on TRM measurement. For qPCR, DNA samples from two individuals (K and F) were placed in 48 wells of a 96-well plate. For each singleplex qPCR assay, we used two plates (one for Telomere and one for Reference gene). For the multiplex qPCR and the Luminex assay, the telomere and the reference genes were assayed from the same well. The coefficient of variation (CV) of the TRM for Luminex (7.2 to 8.4%) was consistently lower than singleplex qPCR (11.4 to 14.9%) and multiplex qPCR (19.7 to 24.3%). In all three qPCR assays the DNA samples in the left- and right-most columns showed significantly lower TRM than the samples towards the center, which was not the case for the Luminex assay (p = 0.83). For singleplex qPCR, 30.5% of the variation in TL was explained by column-to-column variation and 0.82 to 27.9% was explained by sample-to-sample variation. In contrast, only 5.8% of the variation in TRM for the Luminex assay was explained by column-to column variation and 50.4% was explained by sample-to-sample variation. Our novel Luminex assay for TRM had good precision and did not show the well position effects of the sample that were seen in all three of the qPCR assays that were tested.

## Introduction

Telomere length (TL) is a potential biomarker of aging [1–4] and risk for age-related disease [5]. In large scale studies, qPCR is typically used for relative telomere repeat mass (TRM) measurement, primarily due to its low cost and low DNA input requirements compared to gold standard telomere restriction fragment (TRF) using Southern blot. Telomeric repeat sequences pose practical challenge to design PCR primers to amplify the region because of primer dimer formation. In 2002, Richard Cawthon designed a pair of primers with a few intentionally introduced mismatches to avoid primer dimer formation [6], and this primer set has been widely used by many groups in epidemiologic studies to assess the abundance of the telomere sequence as compared to a reference gene. Subsequently, some modifications were made by other groups [4, 5, 7, 8]. These were singleplex (SP) qPCR, where telomere sequence and the reference gene product were amplified in different wells. This leaves some room for error arising from the difference in DNA input in two wells. Addressing this issue, in 2009, Richard Cawthon introduced another important improvement by multiplexing the telomere and reference gene in single well [9]. These multiplex (MP) qPCR primers have been utilized in a large-scale study [10]. However, the study showed that the well position of the sample in the qPCR plate impacts the TRM measurement [10]. Recently we have developed a novel probe-based assay for TRM with signal amplification on the Luminex platform requiring ~50ng DNA [11].

It may be noted that neither the conventional qPCR, nor the Luminex assay directly measures the actual telomere length, rather these assays measure the relative abundance of the telomere repeat sequence–compared to a particular standard sample. The novel Luminex assay is different than the qPCR (both singleplex and multiplex) with respect to the fact that the Luminex assay does not amplify DNA and therefore does not have any issue with DNA amplification–which is an important issue in qPCR. Rather this Luminex assay depends on hybridization of telomeric repeat sequence-specific probes to the telomeric DNA. In this aspect it has similarity to FISH or qFISH, but this assay can only be done in an already extracted DNA sample, not in tissue or in-situ. So it actually measures the number of telomeric repeats and compares that to a “standard” DNA sample. Therefore, this is a relative measure. Without including additional essential calibration steps and appropriate controls, this method cannot be used to measure the absolute telomere length. However, like TRF and qPCR assays, this Luminex assay also estimates only the average telomere length/content in the sample and cannot address heterogeneity in telomere length/content across chromosomes. This Luminex method is well-correlated with the gold standard Southern blot as well as the widely used qPCR method [11, 12].

Here we report, for the first time, a comparison of relative TRM measures obtained from two SP-qPCR, one MP-qPCR, and our novel Luminex assay for TRM, with emphasis on sample positioning.

## Material and Methods

Genomic DNA samples from two individuals (K-DNA and F-DNA) were used to evaluate the well position effect in qPCR with three different primer sets and the Luminex-based assay. DNA was extracted from whole blood using Qiagen FlexiGene DNA kit (Cat#51206). Quantification was done by NanoDrop. The participants gave written consent and the study was approved by the University of Chicago Institutional Review Board (IRB). DNA samples from each person were plated 48 times in a 96-well PCR plate. K-DNA was placed in wells of the upper left quadrant (24 wells) and lower right quadrants (24 wells); F-DNA was placed in the wells of the other two quadrants of each plate (see S1 Fig).

### qPCR assays for TRM

We conducted two different SP-qPCR assays, using two different published primer sets [6, 7]. For each SP-qPCR assay, two plates (one for Telomere and one for Reference gene) were used. The primers and modified thermocycling conditions used for the study are presented in Table 1. For MP-qPCR [9] and the Luminex assay [11], the telomere and reference genes were assayed from the same well. All the qPCR experiments were done using a Bio-Rad CFX96 thermocycler with Quantitect SYBRGreen PCR Kit mastermix (Qiagen Cat # 204145) from the same lot. Input genomic DNA was 25ng/well and 30 μL reaction volumes were used for qPCR.

**Table 1:** PCR primers and thermocycling conditions.

	SP-qPCR set1	SP-qPCR set2	MP-qPCR set3
	(Cawthon 2002)[6]	(Lin 2010)[7]	(Cawthon 2009)[9]
Primers	**Telomere:**	**Telomere:**	**Telomere:**
	tel 1: GGTTTTTGAGGGTGAGGGTGAGGGTGAGGGTGAGGGT	tel1b: CGGTTT(GTTTGG)5GTT	Telg: ACACTAAGGTTTGGGTTTGGGTTTGGGTTTGGGTTAGTGT
	tel2: TCCCGACTATCCCTATCCCTATCCCTATCCCTATCCCTA	tel2b: GGCTTG(CCTTAC)5CCT	Telc: TGTTAGGTATCCCTATCCCTATCCCTATCCCTATCCCTAACA
	**Single-copy gene *(36B4*):**	**Single-copy gene (human beta-globin)**	**Single-copy gene (Albumin):**
	36B4u: CAGCAAGTGGGAAGGTGTAATCC	*HBG1*: GCTTCTGACACAACTGTGTTCACTAGC	*ALB*u: CGGCGGCGGGCGGCGCGGGCTGGGCGGaaatgctgcacagaatccttg
	36B4d: CCCATTCTATCATCAACGGGTACAA	*HBG2*: CACCAACTTCATCCACGTTCACC	*ALB*d: GCCCGGCCCGCCGCGCCCGTCCCGCCGgaaaagcatggtcgcctgtt
DNA quantity	30 μL vol and 25 ng DNA	30 μL vol and 25 ng DNA	25 μL vol and 20 ng DNA
PCR condition	**Telomere:**	**Telomere:**	95°C for 15 min,
	95°C for 15 min	95°C for15 min	2 cycles of
	25 cycle of	25° cycles of	94°C for 15 sec,
	95°C for 15 sec	96°C for 1 sec,	49°C for 15 sec
	54°C for 2 min	54°C for 60 sec,	45 cycles of
	***36B4 gene*:**	***HBG gene*:**	94°C for 15 sec,
	95°C for 15 min	95°C for 15 min	62°C for 10 sec,
	30 cycles of	30 cycles of	74°C for 15 sec,
	95°C for 15 sec	95° C for 15 sec,	84°C for 10 sec,
	58°C for 1 min	58°C for 1 min	88°C for15 sec

For qPCR, it is assumed that the amplicon or PCR product doubles in each PCR cycle. Therefore, the telomere repeat abundance compared to the single gene or the T/S ratio was calculated as [2^CT(telomere)^/2^CT(reference)^]^-1^ = 2^-delta CT^, where CT = cycle threshold, delta CT = CT(telomere)–CT(reference) [6]. For the SP-qPCR assays, the cycle threshold (CT) values of the corresponding wells of the two plates (Telomere plate and Reference gene plate) were used for calculation of the T/S ratio of a sample in a well position. For example, CT values for well A1 of the telomere plate and well A1 of the reference gene plate were used to calculate the T/S ratio of K-DNA in the A1 well position. The relative TRM in the qPCR assays or the qPCR Index was defined as the T/S ratio of a test sample relative to a control sample. Therefore, qPCR index was calculated as 2^-deltaCT(test sample)^/2^-deltaCT(control sample)^ = 2^-delta delta CT^. For this experiment, we considered the average of three central wells (E7, E8 and E9) as the control sample for the calculation of qPCR Index.

### Luminex assay for TRM

In the Luminex assay [11], the telomere and reference genes were assayed from the same well. For this assay, 24 wells (positions A1through H3) were used for standards, and thus DNA from the same two individuals were replicated in 36 wells. The sample layout is shown in S2 Fig K-DNA samples were replicated in upper left well positions A4 through D8 (20 wells) and lower right positions E9 through H12 (16 wells). F-DNA samples were replicated in the remaining 36 wells of the upper right and lower left positions of the 96-well assay plate.

In the Luminex assay, fluorescent Luminex microbeads with capture probes (CP) are used to capture DNA molecules. For each DNA target, two target-specific probe sets are designed: (a) Capture extenders (CE) and (b) Label extenders (LE) and blocker (BL) probes (see S3 Fig). Detailed design is shown in a previous publication [11]. CE has two parts—one part is complementary to the CP sequence on the bead and the other part is complementary to the target DNA sequence that is interrogated. LE has two parts—one is complementary to the target DNA sequence and the other is complimentary to the “pre-amplifier”. The target-specific regions of CE, LE and BL hybridize to contiguous sequences of the target DNA. The preamplifier binds with multiple biotinylated amplifiers. Each amplifier provides multiple hybridization sites for biotinylated label probes that bind Streptavidin R-Phycoerythrin (SAPE) producing fluorescent signals. The signal intensities from the Luminex bead and the conjugated SAPE are read on a Luminex 200 instrument. The signal is reported as median fluorescent intensity (MFI) from at least 100 beads read within 60 second. The MFI is proportional to the number of target sequences in the sample. The probes (both CE and LE) for the telomeric region were designed to target the repeats “TTAGGG”. The 24-mer probe was targeted against 4 repeats–“TTAGGGTTAGGGTTAGGGTTAGGG”. For reference single gene, we used *ALK*. A detail of the assay protocol was described in an earlier publication [11]. All the incubation steps were done in a VorTemp 56 shaking incubator. The assay plate was read in a Luminex 200 instrument. The median fluorescent intensity generated by XPonent software was processed for quantification using Milliplex Analyst software. It may be noted that the MFI is proportional to (a) the number of repeats (which determines the telomere length) and (b) quantity of DNA in the reaction well. Quantification for Telomere and *ALK* (reference gene) was done against the 8-point standard curve (one “no-template control blank” and seven serial dilutions of the “standard” DNA sample from 400 ng to 6.25 ng per well) generated by 5-PL algorithm [13, 14]. The relative telomere repeat mass in the Luminex assay was expressed as Telomere Quantity Index (TQI) and was calculated as Telomere/*ALK* which is normalized for quantity of DNA in a well and it is relative to the “standard” DNA sample [11].

### Statistical Analysis

The coefficient of variation (CV) was calculated as the standard deviation divided by the mean and expressed as a percentage.

We used a 3-way ANOVA model using method of moments [15, 16] for detection of effect of sample positioning.

$$Y_{ijkl}=\mu +{Column}_{i}+{Row}_{j}+{Sample}_{k}+\varepsilon_{ijkl}$$

In this equation, Y is the TRM; where Y_ijkl_ represents the l-th observation on the i-th Column j-th Row k-th Sample; μ is the common effect for the whole experiment.

ε_ijkl_ represents the random error present in the l-th observation on the i-th Column j-th Row k-th Sample. The errors ε_ijkl_ are assumed to be normally and independently distributed with mean 0 and standard deviation δ for all measurements.

The percent explained by a variable was calculated as the sum of squares for that variable divided by the total sum of squares for all the variables in the ANOVA model [17].

## Results

### Precision of the Assays

The CT values from qPCR experiments for all the wells are shown in S1 Table. The quantification data from the Luminex assay, using the 8-point standard curve generated by 5-PL algorithm, for all the sample wells is shown in S2 Table. Coefficient of variation (CV %) of the CT values for the Telomere and the Reference genes in qPCR assays and the Luminex assay are presented in Table 2. It shows that the PCR results were reproducible. However, for all the three primer sets, the CV% of CT values for telomere product was consistently higher (2–3 fold) than the corresponding reference gene product—perhaps representing the fact that it is comparatively difficult to design telomere primers that amplify many regions within the genome. The CV% of TRM measure qPCR Index for K-DNA was 11.42%, 14.90%, 24.30% and 8.48% for SP-qPCR set1, SP-qPCR set2, MP-qPCR and Luminex respectively. Similarly, the CV% for F-DNA was 11.41%, 13.81%, 19.74% and 7.21% respectively.

**Table 2:** Precision of the assays.

	CV% of 48 observations for		CV% of 16 observations for		CV% of 2 observations for	
	K-DNA 48 unique observations	F-DNA 48 unique observations	K-DNA 16 observations of triplicates	F-DNA 16 observations of triplicates	K-DNA 2 observations (24 replicates each)	F-DNA 2 observations (24 replicates each)
CT Telomere_SinglePlex PCR_set1	1.24%	1.26%	0.89%	0.85%	0.26%	0.05%
CT_36B4_SinglePlex PCR_set1	0.31%	0.38%	0.16%	0.28%	0.07%	0.18%
TRM: qPCR Index_SinglePlex PCR_set1	11.42%	11.41%	8.83%	7.48%	3.76%	3.27%
CT_Telomere_SinglePlex PCR_set2	2.28%	2.23%	1.07%	1.43%	0.54%	0.80%
CT_HBG_SinglePlex PCR_set2	0.36%	0.36%	0.30%	0.30%	0.23%	0.02%
TRM: qPCR Index_SinglePlex PCR_set2	14.90%	13.81%	8.85%	8.90%	7.86%	6.02%
CT_Telomere_Multiplex PCR	1.24%	1.26%	0.77%	0.94%	0.39%	0.36%
CT_Alb_Multiplex PCR	0.95%	0.96%	0.68%	0.60%	0.04%	0.20%
TRM: qPCR Index_MultiPlex PCR	24.30%	19.74%	17.44%	14.03%	4.15%	0.51%
	CV% of 36 observations for		CV% of 9 observations for		CV% of 2 observations for	
	K-DNA 36 unique observations	F-DNA 36 unique observations	K-DNA 9 observations of quadruplicates	F-DNA 9 observations of quadruplicates	K-DNA 2 observations	F-DNA 2 observations
Telomere Quantity LUM	5.48%	5.44%	4.36%	3.48%	4.42%	2.46%
ALK Quantity LUM	8.15%	6.80%	3.84%	2.69%	0.89%	0.34%
TRM: TQI_Luminex	8.48%	7.21%	4.76%	3.65%	3.52%	2.12%

Table 2 also shows the effect of “number of observations” considered for the calculation of CV% for qPCR. For example, reducing the number of observation from 48 to 16 to 2 reduces the CV% for K-DNA from 11.42% to 8.83% to just 3.76% respectively. It is to be noted that CV% are typically reported comparing two observations per sample [18, 19]. These two observations may come from two plates (inter-assay CV%) or from the same plate (intra-assay CV%), and for qPCR an observation from one plate typically comes from the mean value of triplicates or quadruplicates of the same sample with or without exclusion of the outlier well.

### Variation of the assays by well position

#### CT values for telomere

**Fig 1:**
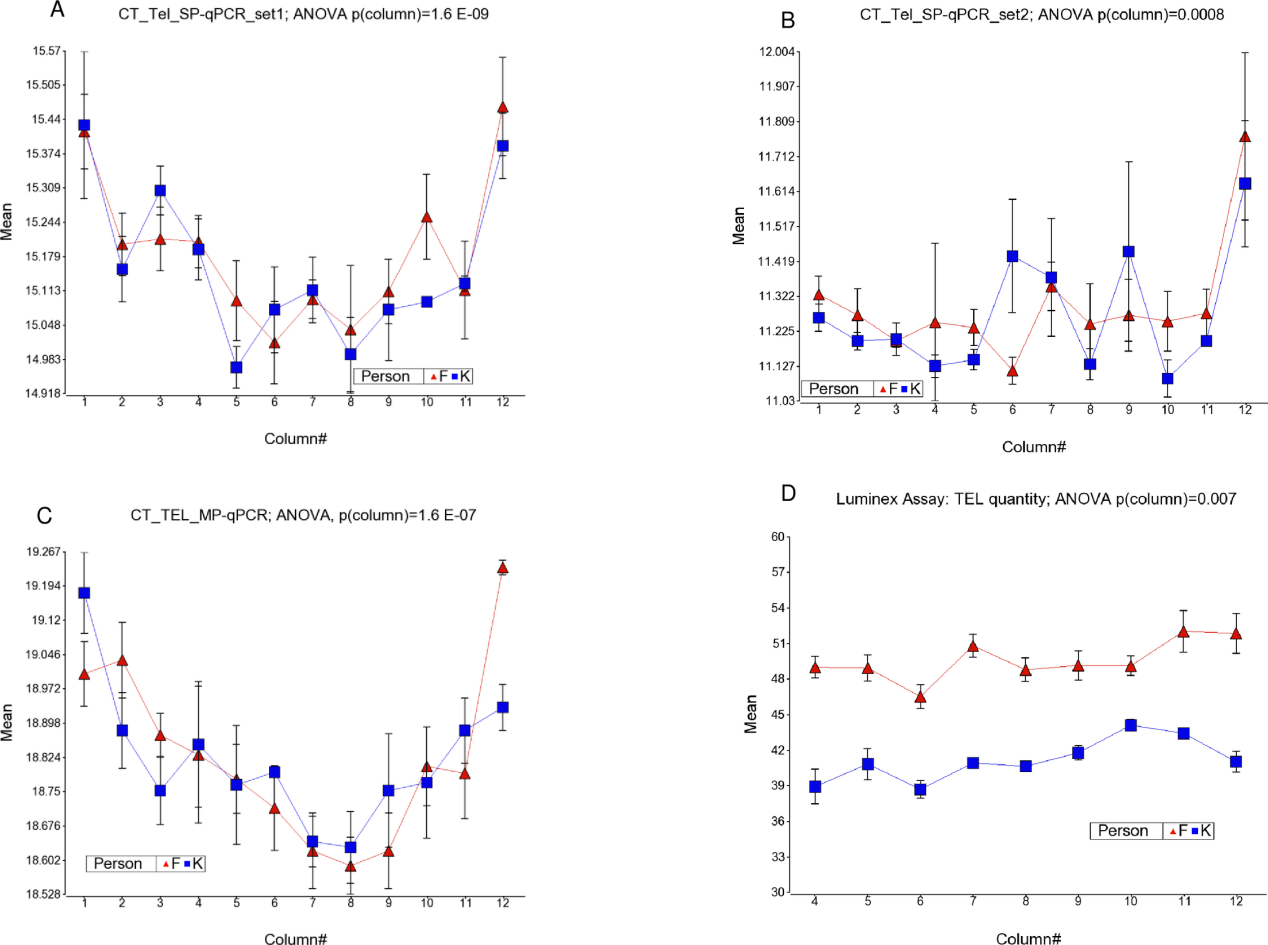
Variation of Telomere products by column. Column number is shown in x-axis and the quantity of Telomere product is shown in y-axis. CT-values of Telomere product (inversely proportional to PCR product quantity) from SP-qPCR-set1, SP-qPCR-set2 and MP-qPCR are shown in fig (A), (B) and (C) respectively. Quantity of Telomere product measured by Luminex assay is shown in (D).

In general, independent of sample and row in the plate, the CT values for telomere product of all three PCR primer sets showed higher values in the left and right most columns (ANOVA, p = 1.6 E-09, p = 0.0008 and p = 1.6 E-07 for SP-qPCR set1, SP-qPCR set2 and MP-qPCR primer sets respectively) indicating less efficient amplification at the edges of the plate (see Fig 1). Similarly, the upper and lower rows of the plate showed higher CT values (indicating lower amplification) compared to the middle rows (ANOVA, p = 0.0007, p = 1.6 E-07 and p = 0.005 for SP-qPCRset1, SP-qPCRset2 and MP-qPCR primer sets respectively) independent of sample and column in the plate.

#### CT values for the reference genes

**Fig 2:**
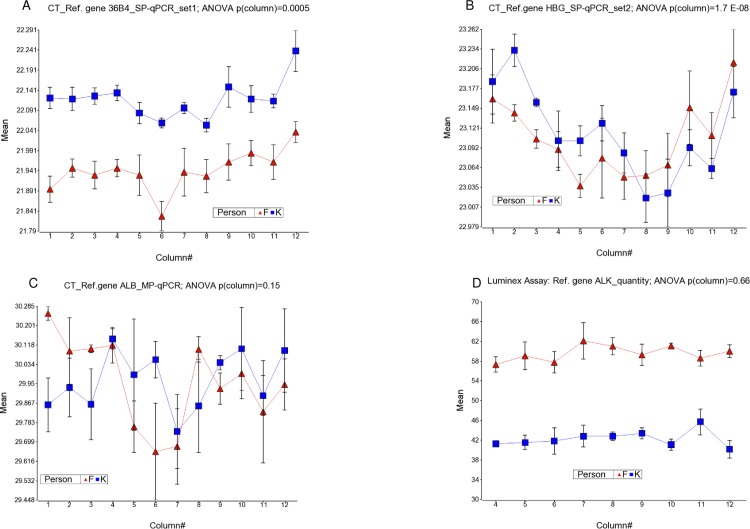
Variation of reference gene products by column. Column number is shown in x-axis and the quantity of reference gene product is shown in y-axis. CT-values of the Reference gene (inversely proportional to PCR product quantity) from SP-qPCR-set1, SP-qPCR-set2 and MP-qPCR are shown in fig (A), (B) and (C) respectively. Quantity of the Reference gene *(ALK)* product measured by Luminex assay is shown in (D).

A similar trend of higher CT values in the left and right most columns (ANOVA, p = 0.0005, p = 1.7 E-08 and p = 0.15 for SP-qPCRset1, SP-qPCRset2 and MP-qPCR primer sets respectively) was also seen for the reference gene for SP-qPCRset1 and SP-qPCRset2 primers, but not for MP-qPCR primers (see Fig 2). Row effect on reference gene product was also statistically significant (ANOVA, p = 0.049, p = 1.6 E-07 and p = 0.15 for SP-qPCRset1, SP-qPCRset2 and MP-qPCR primer sets respectively) for SP-qPCRset1 and SP-qPCRset2 primers, but not for MP-qPCR primers.

#### qPCR Index (Relative Telomere repeat mass)

**Fig 3:**
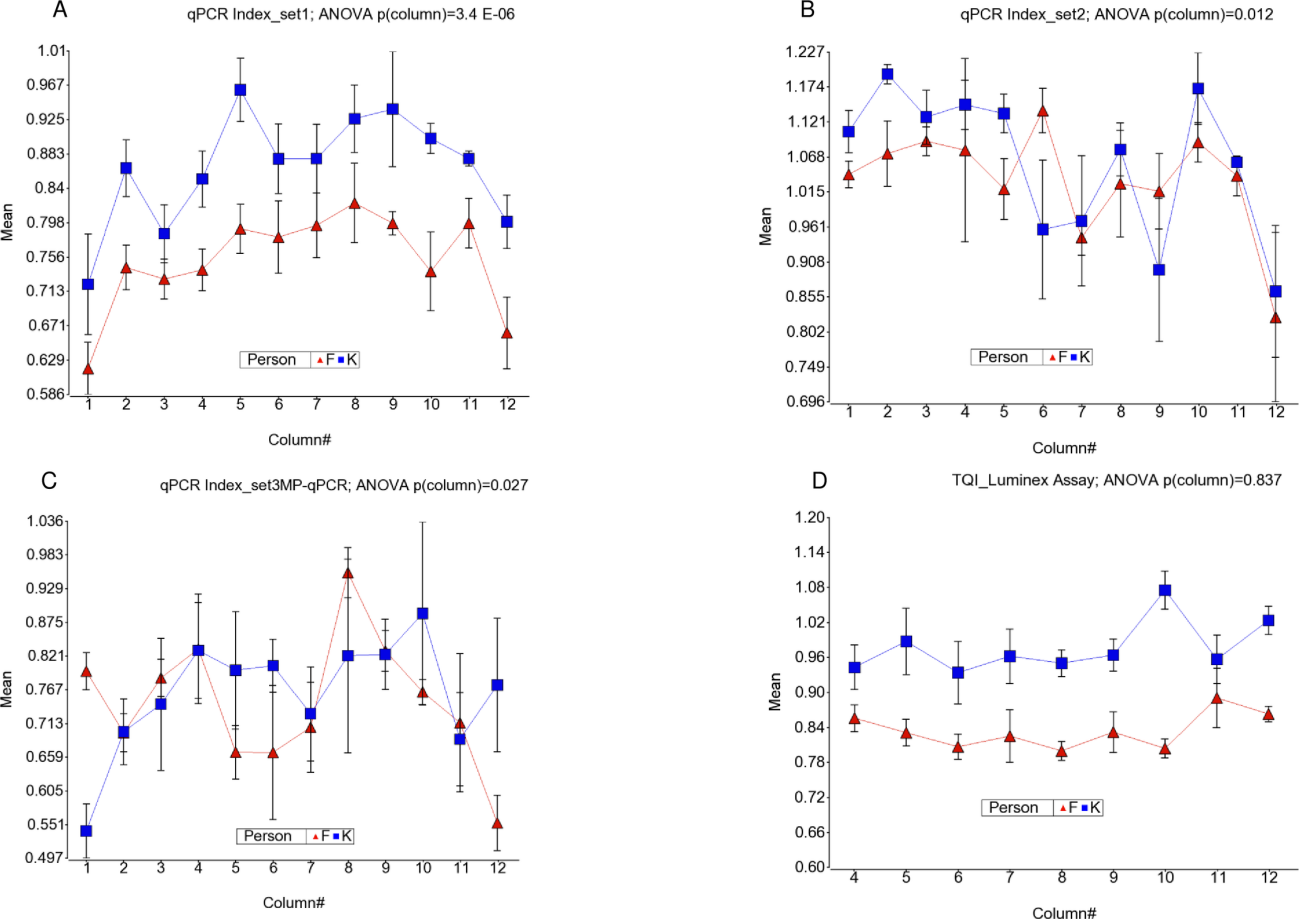
Variation of Relative Telomere repeat mass measures from different assays by column. Column number is shown on the x-axis and the TRM is shown on the y-axis. The qPCR-index measured by SP-qPCR-set1, SP-qPCR-set2 and MP-qPCR are shown in fig (A), (B) and (C) respectively. qPCR index was calculated as 2^-deltaCT(test sample)^/2^-deltaCT(control sample)^ = 2^-delta delta CT^. Telomere Quantity Index (TQI) measured by Luminex assay is shown in (D). TQI was calculated as Telomere/*ALK* which is normalized for quantity of DNA in a well and it is relative to the “standard” DNA sample.

Although both the telomere and reference gene amplification were affected in the same way (i.e., lower amplification in edges compared to central wells), and we considered the corresponding well positions in telomere and reference gene PCR plates for the calculation of the qPCR index, the data showed statistically lower qPCR index in the right- and left-most columns in all three PCR primer sets (ANOVA, p = 3.4x10E-6, p = 0.012, and p = 0.02 for SP-qPCR set1, SP-qPCR set2, and the MP-qPCR assays respectively, see Fig 3). This may be explained by the fact that the telomere qPCR amplification was more strongly affected by the well position than that of the reference gene. This was also suggested by the higher CV for CT (telomere) compared to CT (reference) values. When we ignored the TRM results from the right- and left-most columns for qPCR assays, the statistical differences between the columns was substantially reduced–ANOVA p = 0.05 for set-1, p = 0.04 for set-2, and p = 0.25 for set-3 primers.

#### Novel Luminex Assay: Telomere Quantity Index (TQI)

As mentioned earlier, the relative TRM in the Luminex assay is measured by the ratio of TEL/*ALK*. The effect of positioning the samples in different columns on measured Telomere quantity is presented in the Fig 1(D), and that of the reference gene ALK quantity is shown in Fig 2(D). It was noted that the TQI, measured as Tel/*ALK*, in the Luminex assay did not show statistical difference by column [ANOVA, p = 0.83, see Fig 3(D)] or by row (ANOVA, p = 0.96). The separation of samples from two individuals was also clear in all the columns.

### Relative abundance of “single gene” in qPCR

**Fig 4:**
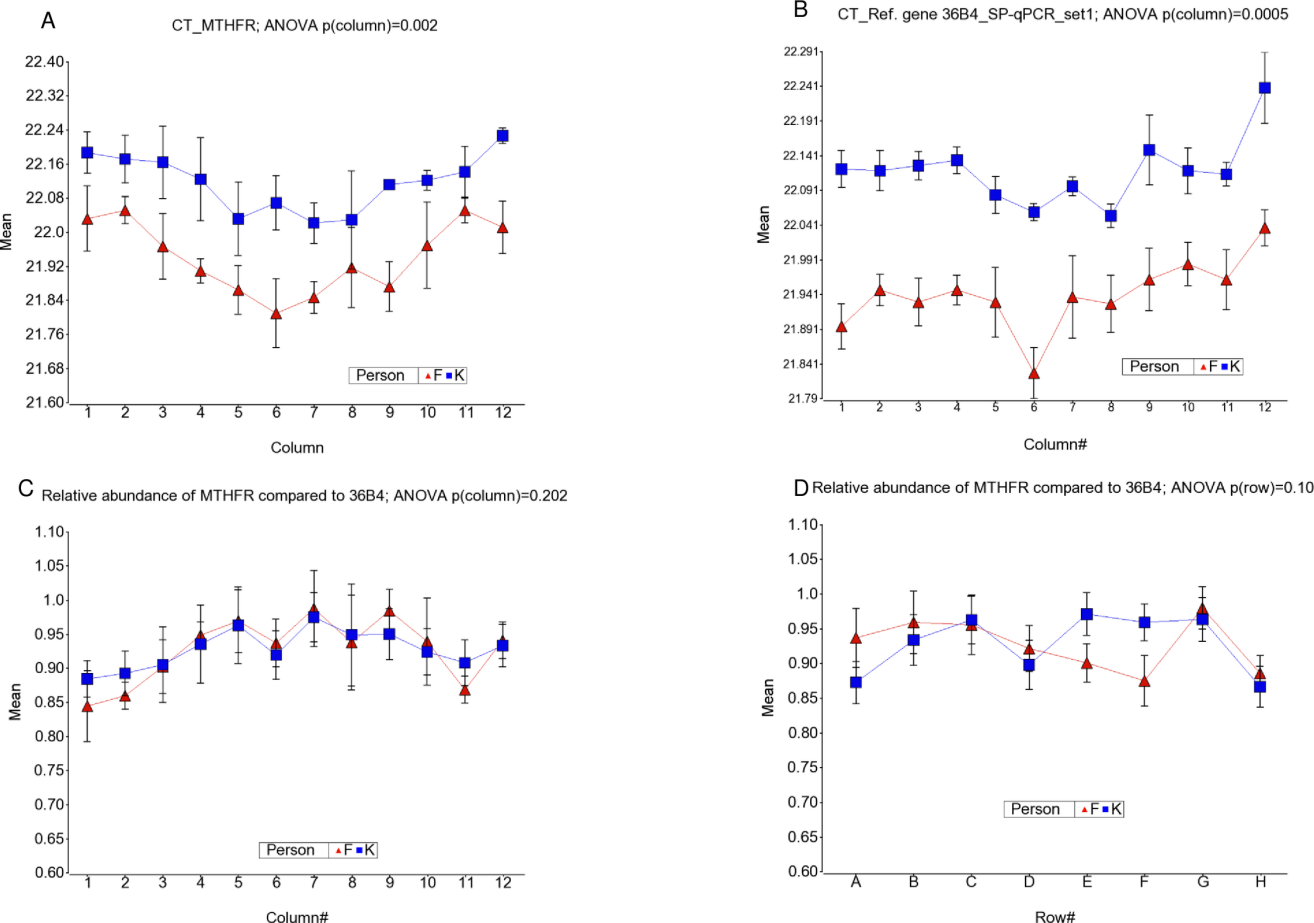
Variation of “single gene” measures from different assays by column or row position. The CT values for the randomly selected single gene MTHFR and the reference gene 36B4 by column are shown in (A) and (B) respectively. The variation of relative abundance of single gene MTHFR (measured as *MTHFR/36B4* ratio) by column and row is shown in (C) and (D) respectively.

We also looked at the ratio of two reference genes (S2/S1 ratio) from two primer sets, instead of T/S. We did not find statistically different results of *HBG*/*36B4* in different columns (ANOVA, p = 0.58) or in rows (ANOVA, p = 0.88). We also amplified a genomic region of the randomly selected *MTHFR* gene. CT values by columns showed similar effects like the single gene in set1 primers–lower amplification in the edges [see Fig 4(A) and 4(B)]. But the index (ratio of *MTHFR* and *36B4*) did not show statistical difference between the columns [ANOVA, p = 0.2; see Fig 4(C)], rows [ANOVA, p = 0.1, see Fig 4(D)] or between samples (ANOVA, p = 0.93). This provides evidence that despite edge effects in a PCR plate, the relative abundance of a “single gene” could be reliably measured with precision using identical well positions from two plates using qPCR method in a heating block based thermocycler like the Bio-Rad CFX96 that was used in this study. But variation in amplification of the telomeric region using the tested PCR primers may not allow that level of precision across the plate as can be achieved for other easy to amplify genomic regions.

### Variation of the Telomere assays explained by “sample-to-sample” variation

We looked at the same data in a different way–how much of the variation in the TRM measures can be explained by (1) “sample-to-sample” variation, (2) “column-to-column” variation and (3) “row-to-row” variation for all the assays. This is important for any population study looking at the difference in TRM in two or more groups. The result of the analysis is presented in Table 3. Among the three qPCR primer sets used in the present study, the set1 assay showed better results in terms of sample-to-sample variation as ~28% of the variation in the qPCR Index from that primer set was accounted for sample-to-sample difference. Performance of set2 (0.82%) and set3 primer (0.19%) was much lower. For the Luminex assay, more than 50% of the variation in TRM was explained by sample-to-sample difference. This may indicate the better suitability of the Luminex assay in comparing two groups in terms of required sample size for the experiment. For example, for a study to find a difference of 5% in TRM among two groups with alpha = 0.05, to have 80% power, the required sample size would be 35 in each group if Luminex assay is used, 55 if SP-set1 qPCR is used, >145 if SP-set2 qPCR is used and >170 if MP-qPCR is used.

**Table 3:** Percentage of variation in the data that can be explained by variation in different factors.

	Percentage of variation explained by			
	column to column	row to row	sample-to- sample	Unexplained
CT Telomere_SinglePlex PCR_set1	49.62%	13.77%	0.48%	36.13%
CT_*36B4*_SinglePlex PCR_set1	14.21%	4.70%	58.04%	23.05%
TRM: qPCR Index_SinglePlex PCR_set1	30.54%	7.84%	27.99%	33.64%
CT_Telomere_SinglePlex PCR_set2	31.37%	8.58%	0.25%	59.79%
CT_*HBG*_SinglePlex PCR_set2	38.48%	29.21%	0.31%	32.00%
TRM: qPCR Index_SinglePlex PCR_set2	30.58%	5.08%	0.82%	63.52%
CT_Telomere_Multiplex PCR	45.17%	11.20%	0.01%	43.61%
CT_*Alb*_Multiplex PCR	14.89%	9.27%	0.03%	75.81%
TRM: qPCR Index_MultiPlex PCR	17.52%	19.53%	0.19%	62.76%
Telomere Quantity Luminex	8.94%	1.36%	74.34%	15.36%
*ALK* Quantity Luminex	1.27%	0.19%	84.32%	14.22%
TRM:TQI_Luminex	5.78%	1.40%	50.37%	42.44%

### Validation of TRM of these two DNA samples by TRF

The telomere length of the K-DNA and F-DNA samples were measured in the past by Southern blot of the TRF method [20] in the Aviv lab during the Luminex assay development. The TL of K-DNA was 9.12 kb and that of F-DNA was 8.81 kb. So the telomere length was ~3% longer in K-DNA compared to F-DNA. It may be noted that in the Luminex assay and SP-qPCR with set1 primers, the TRM of K-DNA was persistently higher than F-DNA in all the columns (see Fig 3), but this difference in TRM was not clearly seen in SP-qPCR with set2 or the MP-qPCR with set3 primers.

## Discussion

To our knowledge, this is the first study specifically designed to examine the effect of well position on TRM measurement by qPCR using three different published primer sets and by the Luminex assay. All the three qPCR primer sets used in this study efficiently amplified the telomere product. Well effect on TRM measurement by MP-qPCR was only recently reported [10], although the concern was implicitly acknowledged through the randomization of well positions across replicates in previous studies [9, 21]. The recently published, largest study addressing the TRM measurement using qPCR also used randomization [4]. Our study also allowed us to evaluate the precision of the assays. The edge-effect in qPCR is not uncommon (http://www.protocol-online.org/biology-forums-2/posts/17528.html). If the PCR primer efficiency of the telomere and the reference gene is the same, then multiplexing the telomere and the reference gene reactions in a single well or comparing the CT values from the same well location of two plates (one for target and one for reference gene) may increase the precision and accuracy of the relative abundance measurement. This would be true even in the presence of an edge effect due to well-to-well thermal difference in heating block of the thermocycler. Perhaps that is why when we looked at the relative abundance of one of the reference genes or another randomly picked gene (*MTHFR*), we did not see a significant well-position effect. Our experiments showed statistically significant lower amplification of telomere product at the edges compared to center wells in the qPCR assay, and this significantly affects the measured relative abundance of TRM (or the qPCR index). The Luminex assay did not show any significant effect of well position on the measured relative TRM. It may be noted that for the Luminex assay, all the incubations were done in a shaking incubator, not in a thermocycler.

We also evaluated the precision of the qPCR and Luminex assays. In contrast to primers for a reference gene that amplify a unique genomic region, the telomeric primers amplify multiple regions in the genome, giving rise to a large number of amplicons of different sizes. The CV% of CT values for telomere products are two to three times higher than those for the single gene products for all the three primer sets, reflecting the variability of telomere product. For the calculation of relative abundance of telomere (ratio = 2^-delta CT^), even a slight difference in delta CT may cause relatively large changes in the relative telomere abundance. Table 2 clearly shows that phenomenon—although most of the CV% for the CT-values were less than 1.5% (for telomere) and less than 1% (for reference gene), the CV% of qPCR Telomere Index (which is the final estimate for TRM) varied widely from 0.5% to 24% depending on primer set and number of observations used for the calculation. For reporting qPCR-based Telomere assays, usually the mean of triplicate or quadruplicate samples are used to obtain a single observation, and authors report CV% from paired observations. Our qPCR CV% results calculated from two observations (each from a mean of 24 replicates) or from 16 observations (each from triplicate) are not different from published reports [4, 19]. Unlike qPCR, the novel Luminex assay does not necessarily require replicate samples for TL measurement. Even in 36 observations, the CV% was <9%, which is comparable to reported CV% for qPCR using replicates.

The fact that we observed lower PCR yield for multiple PCR products at the edges of the plate (specially right and left columns) may indicate differential heating of the heating block. However, it may be noted that the well-effect was not found for relative abundance measurement of reference gene or *MTHFR* using qPCR method; it was found only for telomere product. Therefore, this may indicate problems with the telomere assay itself (difficult primer design / possible complexity of telomeric region, amplification with PCR primers), and the problem is not of the qPCR platform. It may be noted that when we ignored the TRM results from the right- and left-most columns for qPCR assays, the statistical differences between the columns were much reduced. This prompts us to suggest that for the qPCR telomere assay, it may be a good practice to avoid putting samples in the edge-wells. In contrast, the Luminex assay does not show statistically significant impact of well position on the telomere measurement. The Luminex assay was also better in differentiating among different individuals/samples and may provide an advantage for sample size requirement in study design.

The Luminex assay for telomere correlates well with the gold standard TRF. We have recently reported a blinded comparison of this Luminex assay and TRF by Southern blot—the gold standard [12]. Luminex and Southern blot measurements for the same 50 DNA samples were taken in two independent laboratories (Ahsan Lab and Aviv Lab); each sample was measured twice, several months apart. The correlation (r) between Southern blot and Luminex was 0.65 in round-1 and 0.75 in round-2 [12].

One of the drawbacks of the current study is the use of only one qPCR platform–BioRad CFX-96, and therefore we cannot comment on whether the well-effect seen in our study may hold true for other block based thermocyclers. However, other studies have documented well-effect in other qPCR instruments like iCycler [10] and LightCycler 480 [22].

### Limitation of qPCR assay for TRM

Like TRF, the qPCR assays also estimates only the average TRM in the sample and cannot address heterogeneity and it renders relative values and cannot provide absolute telomere length values in kilo bases (kb). A critical evaluation of qPCR Telomere size techniques was recently published [23]. The precision of qPCR varies substantially among prior studies, with CVs of <7% [18, 24], 7%-11% [25], ~15% [26] and even >25% being reported [27]. A recent study across six labs reported substantial heterogeneity in per-sample intra-batch CVs (0%-31%) and inter-batch CVs (0.2%-28%) [19]. In connection with higher inter-assay CV, a larger sample size is needed when using qPCR in comparison with using TRF or STELA [28]. The major methodological variables of the qPCR estimation of telomere size that often create difficulties in data interpretation includes (a) DNA isolation [29], (b) data verification and (c) statistical analysis [23]. It has been suggested that southern blots need to be performed on a subset of qPCR samples for mean telomere size and there is a need for greater transparency in discussing the limitations of the qPCR data [23]. A recent study suggests that a modification of the original SP-qPCR with two-plate T/S assay was more compatible than the recently developed multiplex qPCR in single-plate assay, and that choice of hot-start Taq polymerase and intercalating dye were critical factors [30]. Keeping the mastermix (Taq polymerase, dNTPs, buffer) same for all three tested qPCR assays, our present study shows the effect of primer choice as well as the “well-position effect” of the samples in the qPCR plate on the TRM measurement.

### Limitations of Luminex assay

Like TRF and qPCR assays, this Luminex assay also estimates only the average TRM in the sample and cannot address heterogeneity. Currently, the Luminex assay generates an estimate of relative TRM. It is possible to generate absolute telomere length in kb only by calibrating against a DNA sample with known absolute telomere length. We acknowledge that calibration with TRF measurement may not be accurate because of the fact that TRF takes the subtelomeric region into account; and by design, the Luminex assay uses hybridization probe specific to the telomeric repeats only.

## Conclusion

Our novel Luminex assay offers an alternative method for average relative TRM measurement in a sample with good precision and without significant “well position effects” for large-scale study.

## Supporting Information

S1 Fig Sample layout for qPCR assays. (DOC)

S2 Fig Sample layout for Luminex assay. (DOC)

S3 Fig Schematic diagram of the Luminex assay. A, hybridization step; B, addition of pre-amplifier and amplifiers; C, binding of SAPE–producing fluorescent signals.
(PPT)

S1 Table qPCR data for Telomere and reference gene for calculation of qPCR index. (XLS)

S2 Table Quantification data for the Luminex assay for calculation of Telomere Quantity Index (TQI). Quantification for Telomere and *ALK* (reference gene) was done against the 8-point standard curve generated by 5-PL algorithm. Telomere Quantity Index (TQI) and was calculated as Telomere/*ALK* which is normalized for quantity of DNA in a well and it is relative to the “standard” DNA sample
(XLS)
