# Supplementary material for: Novel Luminex Assay for Telomere Repeat Mass Does Not Show Well Position Effects Like qPCR
Source: PLoS One. 2016 May 16;11(5):e0155548. doi: 10.1371/journal.pone.0155548 (PMC4868509; doi:10.1371/journal.pone.0155548)
Supplement: S2 Fig — (DOC) [file pone.0155548.s002.doc]

S2: Fig: Layout of DNA samples for Luminex assay:

|  | 1 | 2 | 3 | 4 | 5 | 6 | 7 | 8 | 9 | 10 | 11 | 12 |
| --- | --- | --- | --- | --- | --- | --- | --- | --- | --- | --- | --- | --- |
| A | Blank | STD2 | STD5 | K | K | K | K | K | F | F | F | F |
| B | Blank | STD3 | STD5 | K | K | K | K | K | F | F | F | F |
| C | Blank | STD3 | STD6 | K | K | K | K | K | F | F | F | F |
| D | STD1 | STD3 | STD6 | K | K | K | K | K | F | F | F | F |
| E | STD1 | STD4 | STD6 | F | F | F | F | F | K | K | K | K |
| F | STD1 | STD4 | STD7 | F | F | F | F | F | K | K | K | K |
| G | STD2 | STD4 | STD7 | F | F | F | F | F | K | K | K | K |
| H | STD2 | STD5 | STD7 | F | F | F | F | F | K | K | K | K |
