# Supplementary figures and images for: Novel Luminex Assay for Telomere Repeat Mass Does Not Show Well Position Effects Like qPCR
Source: PLoS One. 2016 May 16;11(5):e0155548. doi: 10.1371/journal.pone.0155548 (PMC4868509; doi:10.1371/journal.pone.0155548)

## Slide 1
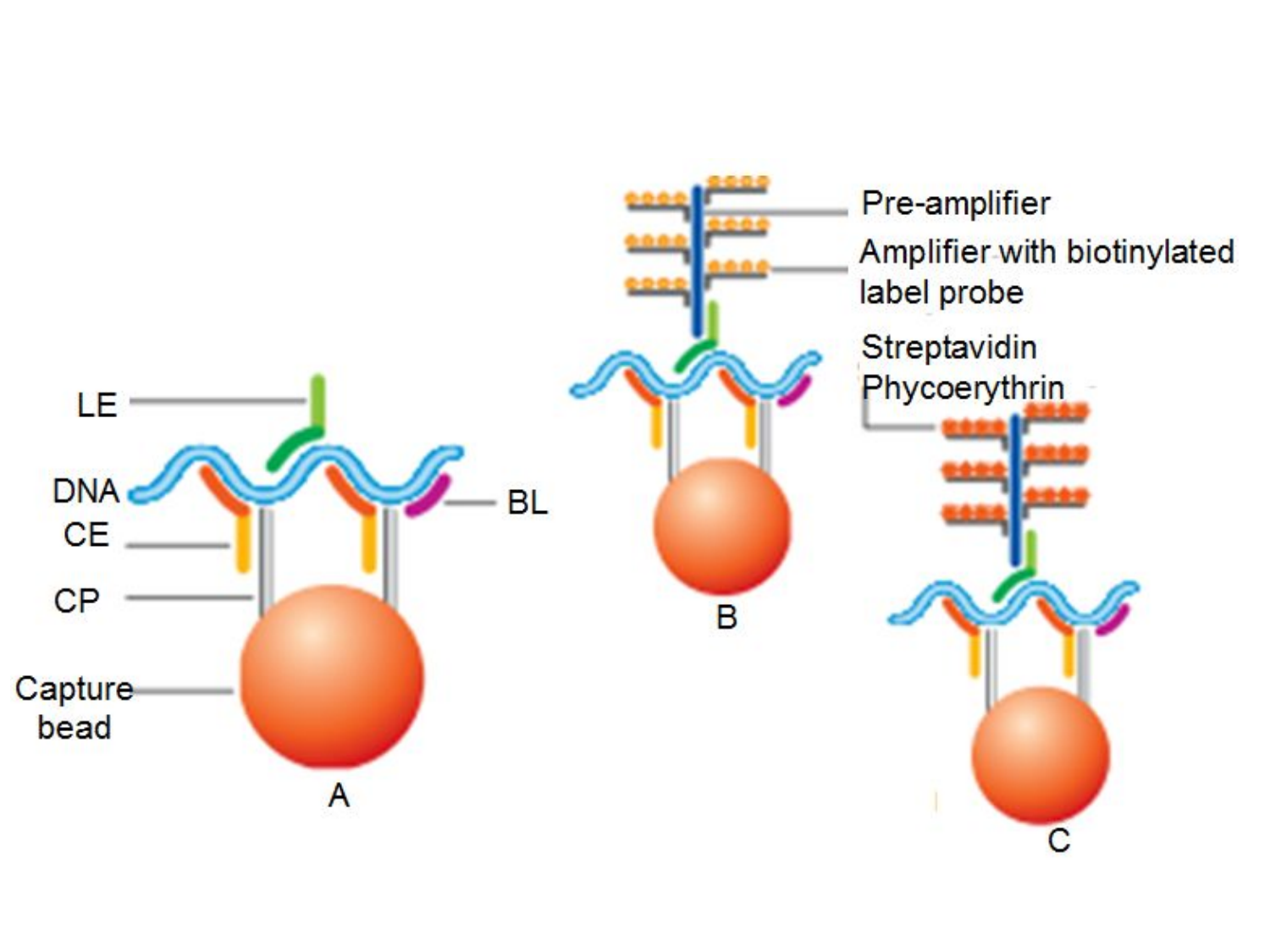

Supplement: S3 Fig — A, hybridization step; B, addition of pre-amplifier and amplifiers; C, binding of SAPE–producing fluorescent signals. (PPT) [file pone.0155548.s003.ppt]
